# The effect of esketamine on emergence delirium or agitation in children after anesthesia-a systematic review and meta-analysis

**DOI:** 10.1186/s12871-026-03748-5

**Published:** 2026-03-16

**Authors:** Wei Yang, Ling Zhou, Yun Xue, He Huang

**Affiliations:** 1 Department of Anesthesiology, Chongqing General Hospital, Chongqing University, Chongqing, China; 2 Department of Anesthesiology, People’s Hospital of Pidu District, Sichuan Province, Chengdu City, China; 3 Department of Anesthesiology, The Second Affiliated Hospital of Chongqing Medical University, Chongqing, China

**Keywords:** Esketamine, Pediatric, Agitation, Delirium, Meta-analysis

## Abstract

**Background:**

This systematic review and meta-analysis aim to evaluate esketamine on emergence delirium or emergence agitation (ED/EA) in pediatric children in contrast to the control group.

**Methods:**

The PubMed, Web of Science, and Cochrane Library databases were systematically searched until August 2024, employing inclusion criteria focused on studies involving children under the age of 18 comparing esketamine to control groups. The exclusion criteria encompassed participants aged 18 years and older, reviews or meta-analyses, fundamental animal studies, articles published solely in abstract form, letters to the editor, case reports, editorial notes, methodological protocols, and publications not in English.

**Results:**

A total of 1142 children were included in ten randomized controlled trials (RCTs) and one cohort study, revealing that esketamine significantly reduces the risk of ED/EA in pediatric patients following surgery when compared to the control group (OR = 0.70, 95% CI: (0.51, 0.97), I^2^ = 80%, *P* for effect = 0.03). Subgroup analysis indicated that low-dose esketamine was particularly effective in mitigating postoperative ED or EA (OR = 0.33, 95% CI: (0.20, 0.53), I^2^ = 19%, *P* for effect < 0.00001), as well as reducing postoperative pain scores (SMD = -1.30, 95% CI: (-2.28, -0.32), I^2^ = 95%, P for effect = 0.009). Furthermore, it did not elevate the incidence of postoperative nausea and vomiting (PONV) (OR: [0.44–1.19], I^2^ = 27%, *P* for effect = 0.20).

**Conclusions:**

In comparison to the control group, esketamine significantly decreases the incidence of emergence delirium or emergence agitation (ED/EA) in pediatric patients, with a markedly lower heterogeneity (I^2^) observed at lower dosages(esketamine < 0.5 mg/kg). Additionally, esketamine effectively mitigates postoperative pain scores in children without elevating the occurrence of PONV.

**Trial registration:**

Prospero registration ID:CRD42024583287. Link:PROSPERO (york.ac.uk).

**Supplementary Information:**

The online version contains supplementary material available at 10.1186/s12871-026-03748-5.

## Introduction

Pediatric emergence delirium, also known as emergence agitation(ED/EA), is a multifaceted psychomotor disorder characterized by perceptual disturbances, delusions, and subsequent disorientation [1]. This condition manifests through aimless movements, restlessness, incoherence, and inconsolability [2]. The underlying mechanisms of ED/EA are unclear. Nevertheless, age, type of surgery, and the use of sevoflurane anesthesia have been identified as independent risk factors. Studies indicate that the incidence of ED/EA following general anesthesia in children ranges from 10 to 80% [3]. Furthermore, the occurrence of these symptoms can lead to self-harm among children, prolong hospitalization duration and costs while diminishing parental satisfaction [4]. Therefore, it is imperative to investigate strategies for mitigating the incidence of delirium or irritability.

Numerous meta-analyses have suggested that ketamine may be moderately effective in mitigating the incidence of ED/EA in pediatric patients [5–7]. Esketamine, as the S + isomer of ketamine, possesses twice the potency of ketamine. It stands out for its mild respiratory depression and also provides more reliable analgesia and sedation with fewer mental side effects compared to ketamine, especially suitable for short-term surgical anesthesia in children [8]. Furthermore, its sympathomimetic properties offer a counterbalance to the hemodynamic inhibition of propofol, thus reducing the risk of respiratory depression and cardiovascular depression during sedation [9]. Although several trials have examined esketamine’s effect on ED/EA, results remain inconsistent [10–14], and no prior meta-analysis has evaluated dose-specific effects.

## Methods

This systematic review and meta-analysis was conducted in accordance with the guidelines established by the Preferred Reporting Items for Systematic Reviews and Meta-Analyses (PRISMA) [15]. The protocol for this systematic review was submitted in advance to the International Prospective Register of Systematic Reviews (PROSPERO), under registration ID: CRD42024583287.

### Search strategy and study selection

Searches were conducted in the Cochrane Library, PubMed, and Web of Science from their inception until August 2024. The search strategy was as follows: (“esketamine” OR “L-Ketamine” OR “(-)-Ketamine” OR “S-Ketamine” OR “(S)−2-(o-chlorophenyl)−2-(methylamino)cyclohexanone”) AND (“emergence agitation” OR “Delirium, Emergence” OR “Agitated Emergence” OR “Emergence, Agitated” OR “Emergence Agitation” OR “Agitation, Emergence” OR “Agitations, Emergence” OR “Emergence Excitement” OR "Excitement, Emergence" OR "Postanesthetic Excitement" OR "Excitement, Postanesthetic" OR "Anesthesia Emergence Delirium" OR "Delirium, Anesthesia Emergence"OR "Emergence Delirium, Anesthesia"OR "Postoperative Delirium"OR "Delirium, Postoperative"OR "Post-Operative Delirium"OR "Delirium, Post-Operative"OR "Post Operative Delirium"). All studies examining the effects of esketamine compared to other drugs (placebo or alternative analgesics and sedatives) on agitation or delirium in pediatric patients were deemed eligible for this meta-analysis; thus we did not impose restrictions on control drug terminology or study designs. The inclusion criteria were as follows: (1) participants aged < 18 years; (2) management involving esketamine alongside placebo or other analgesics and sedatives. The exclusion criteria included: (1) participants aged ≥ 18 years; (2) reviews or meta-analyses; (3) basic animal research; (4) articles published solely as abstracts, letters, case reports, editorials notes methods or protocols; and (5) non-English language publications.

### Literature quality evaluation and data extraction

Quality evaluation (YW and ZL) and data extraction (YW and ZL) were conducted independently by two researchers. The authors meticulously documented the characteristics and outcomes of the experiments, followed by a thorough cross-checking process to resolve any discrepancies through discussion.

The risk of bias in randomized controlled trials (RCTs) was assessed using the Cochrane Collaboration's Risk of Bias Assessment Tool, which encompasses seven criteria: random sequence generation, allocation concealment, blinding of participants and personnel, blinding of outcome assessment, incomplete outcome data, selective reporting, and other factors. For cohort studies, bias risk was evaluated using the Newcastle–Ottawa Quality Assessment Scale (NOS), consisting of three domains: selection, comparability, and exposure. Studies receiving four stars in the selection domain, two stars in comparability, and three stars in exposure were classified accordingly; those accumulating seven or more stars were deemed high quality while those with six stars were categorized as medium quality; studies with fewer than six stars were considered lown quality.

The data extraction included the following elements: (1) authors; (2) publication year; (3) total number of participants per study; (4) age range of all participants; (5) country of publication; (6) type of surgery performed along with anesthesia method used; (7) dosage and administration details for esketamine; and (8) incidence rates for emergency department visits or adverse events following sedation or general anesthesia.

### Data analysis

Statistical analyses were conducted utilizing Review Manager 5.4 software (Cochrane Collaboration, Oxford, UK) and Stata version 16.0 (Stata Corp, College Station, TX, USA). When the original data were represented as continuous variables, a meta-analysis was performed using the Standardized Mean Difference (SMD). This methodology facilitates the aggregation of results measured with different tools and is particularly well-suited for pediatric pain assessment. In cases where raw data were presented as medians (interquartile ranges), we converted them to means (standard deviations) using a conversion formula developed by the University of Hong Kong (www.math.hkbu.edu.hk/~tongt/papers/median2mean.html). Notably, if this dataset did not conform to a normal distribution, it was excluded from the meta-analysis. For dichotomous variables, outcome incidence analysis was executed using odds ratios (ORs) calculated via the Mantel–Haenszel method with 95% confidence intervals (CIs).

Heterogeneity was assessed employing I^2^ statistics, which describe the percentage of variability in effect estimates (OR or SMD) attributable to heterogeneity rather than sampling error. Values of I^2^ < 40%, 40–60%, and > 60% indicated low, moderate, and high heterogeneity respectively. If I^2^ > 50% or a *P*-value for heterogeneity < 0.1 was identified, random-effects model analysis was applied; conversely, if I^2^ < 50% or a *P*-value for heterogeneity ≥ 0.1 emerged, fixed-effects model analysis was utilized [16].

To address issues related to high heterogeneity (I^2^ > 40%), we employed subgroup analyses or systematically removed studies one at a time. Based on potential risk factors identified in the literature review process, meta-regression analyses were conducted on groups exhibiting I^2^ > 40%. Meta-regression focused on risk factors yielding *P*-values < 0.05; conversely, when all risk factor *P*-values equaled or exceeded 0.05, an article-by-article elimination approach was adopted.

## Results

### Literature search results and features

**Fig. 1:**
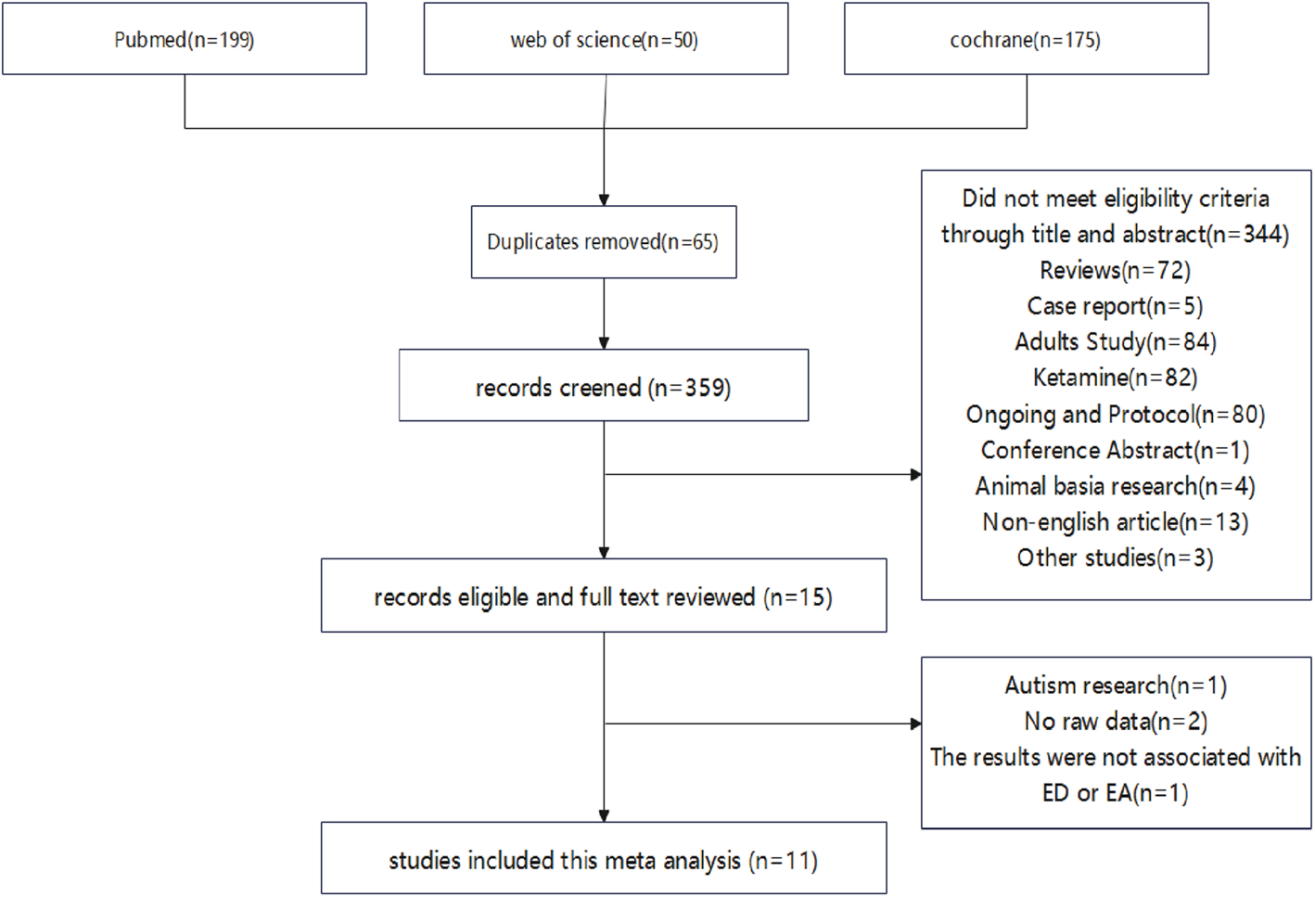
Research screening flow chart

The process of literature screening that meets the conditions were shown in Fig. 1. We obtained 199 trials from Pubmed database, 50 from Web of sciences database and 175 from cochrane database. Sixty-five trials were removed due to duplicates. Three hundred and thirty-four trials were excluded by reading abstracts and titles. The remaining fifteen trials were read through in full, of which one was about a study on children with autism, two were unable to obtain raw data, and one had no correlation with ED/EA. Finally, eleven trials including 1142 patients were included in this meta-analysis [10–12, 14, 17–23].

Table 1 presented the basic information of all included studies, including ten randomized controlled trials(RCTs) and one cohort study [14]. It was not difficult to find that differences in study methods, age of included participants, and usage and dosage of esketamine were the main causes of clinical heterogeneity. As can be seen from the table, the overall age range of the participants included in this meta-analysis ranged from 3 months to 12 years old, and the types of surgery included Otorhinolaryngology surgery, urology surgery, ophthalmic surgery, lower abdomen and perineal surgery, etc. The usage of esketamine included intravenous injection, nasal spray and postoperative PCA analgesia. Dosage can be divided into high dose and low dose. For outcome indicators, we mainly collected PAED score, incidence of PAED (N,%), postoperative pain score, postoperative nausea, vomiting, drowsiness and other complications.

**Table 1:** The basic information of all included trials

Study	N	Age	Country/Center	Procedures	Anesthesia	Esketamine	Control Outcome	Outcome
Chen 2023 [12, 24]	10	3–7 years	China/Single	Tonsillectomy and/or adenoidectomy	General anesthesia (sevoflurane)	0.25 mg/kg intravenous at the end of anesthesia	Placebo (normal saline)	①②③④
Han 2024 [17]	84	2–6 years	China/Two-center	Otorhinolaryngology Adenoidectomy Urology Circumcision Ophthalmic Strabismus Chalazion	General anesthesia (propofol)	General anesthesia was induced with intravenous esketamine 0.25 mg/kg and propofol	General anesthesia was induced with intravenous propofol	①②③④
Li 2022 [11]	80	2–7 years	China/Single	Tonsillectomy	General anesthesia (sevoflurane)	0.25 mg/kg intravenous at the end of surgery	Placebo (normal saline)	②④
Liu 2022 [10]	90	3–6 years	China/Single	Strabismus	General anesthesia (sevoflurane and propofol)	Before induction, nasal spray esketamine 0.5 mg/kg Before induction, nasal spray esketamine 1 mg/kg	Before induction, nasal spray normal saline	①②③④
Xing 2024 [19]	74	2–6 years	China/Single	Dental caries treatment	General anesthesia (sevoflurane)	0.5 mg/kg esketamine and 4ug/kg dexmedetomidine intranasal administration	4ug/kg dexmedetomidine intranasal administration	①②③④
Xu 2023 [25]	154	3 months to 6 years	China/Single	hypospadias	General anesthesia (sevoflurane) and caudial epidural block	PCIA: an initial dose of esketamine 0.3 mg/kg (2 ml) and a maintainance dose of esketamine 0.15 mg/kg/h	PCIA: an initial dose of hydromorphone 0.02 mg/kg (2 ml) and a maintainance dose of hydromorphone 0.01 mg/kg/h	①③④
Zhang 2022 [22]	66	6 months to 10 years	China/Single	Totally implantable venous access port	General anesthesia	Target-controlled infusion of propofol 4ug/ml and esketamine 0.5 mg/kg as induction, and target-controlled infusion of propofol 3-4ug/ml as maintenance	Propofol 2 mg/kg, cisatracurium 0.2 mg/kg, and fentanyl 3ug/kg as induction, inhaled sevoflurane with 0.8 MAC as maintenance	①④
Zhang 2023 [23]	90	3–7 years	China/Single	Fiberoptic bronchoscopy	General anesthesia and local anesthesia	Before induction, 0.5 mg/kg esketamine was intravenously injected Before induction, 0.75 mg/kg esketamine was intravenously injected	Before induction, normal saline was intravenously injected	①③④
Xie 2024 [20]	76	2–12 years	China/Single	Urology	General anesthesia (sevoflurane)	0.3 mg/kg esketamine during anesthesia induction, PCIA:1.0 mg/kg of esketamine and 0.1 mg/kg of hydromorphone Equal volume of saline during anesthesia induction, PCIA:1.0 mg/kg of esketamine and 0.1 mg/kg of hydromorphone	PCIA: administered at a dose of 0.1 mg/kg hydromorphone PCIA: administered at a dose of 0.15 mg/kg hydromorphone	②④
Chen Sai 2024 [14]	230	2–7 years	China/Single	Otorhinolaryngology Urinary	General anesthesia	A single bolus of esketamine (0.46 mg/kg: average dose) for general anesthesia	Placebo	①②③

Outcome measures included: ① PAED scores; ② Emergence agitation (N, %); ③ Pain scores; ④ Postoperative complications included (drowsiness, postoperative)

### Bias risk assessment

**Fig. 2:**
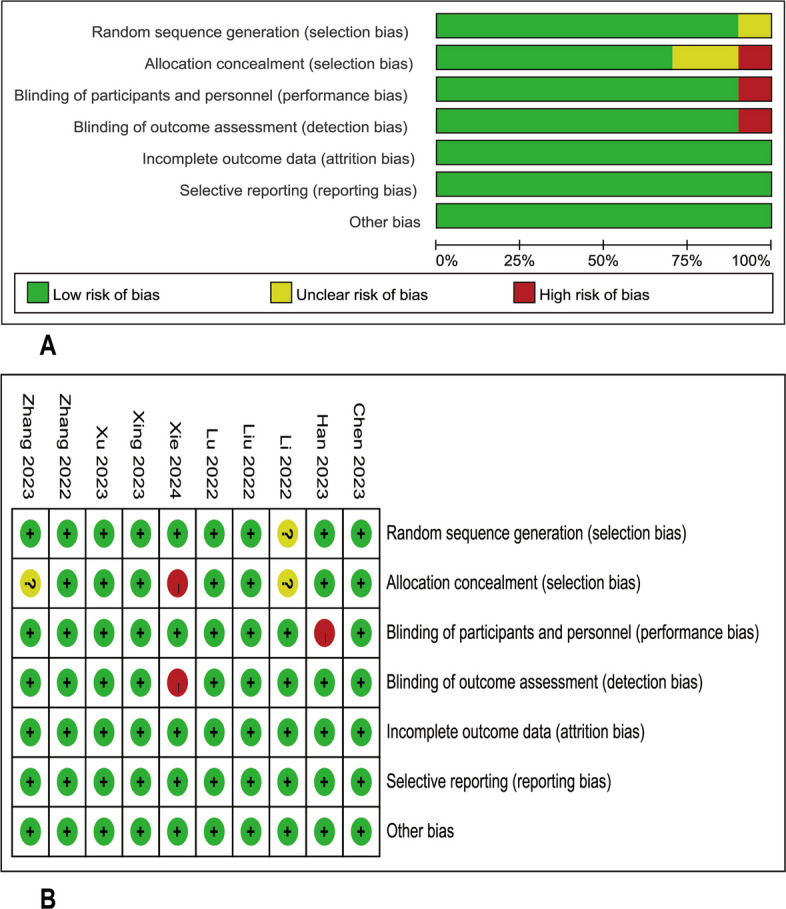
Literature quality evaluation. **A** Risk of bias graph; **B** risk of bias summary

Cochrane Collaboration Risk of Bias Assessment was applied to all ten RCTs trials included in this meta-analysis, and the assessment results were shown in Fig. 2. One cohort study was evaluated using the Newcastle-Otawa Quality Assessment scale and was rated eight stars.

### Main results of meta-analysis

#### Esketamine for ED/EA

**Fig. 3:**
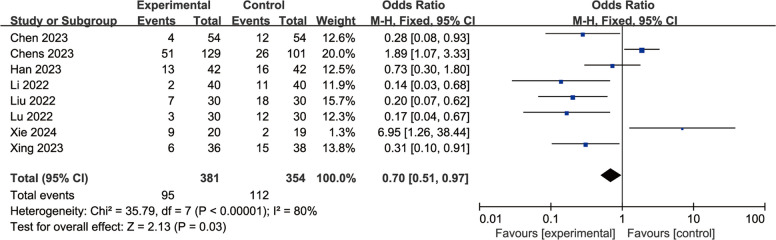
Comparison of pediatric EA/ED between esketamine and the control group (Fixed effects) from RevMan 5.4

**Fig. 4:**
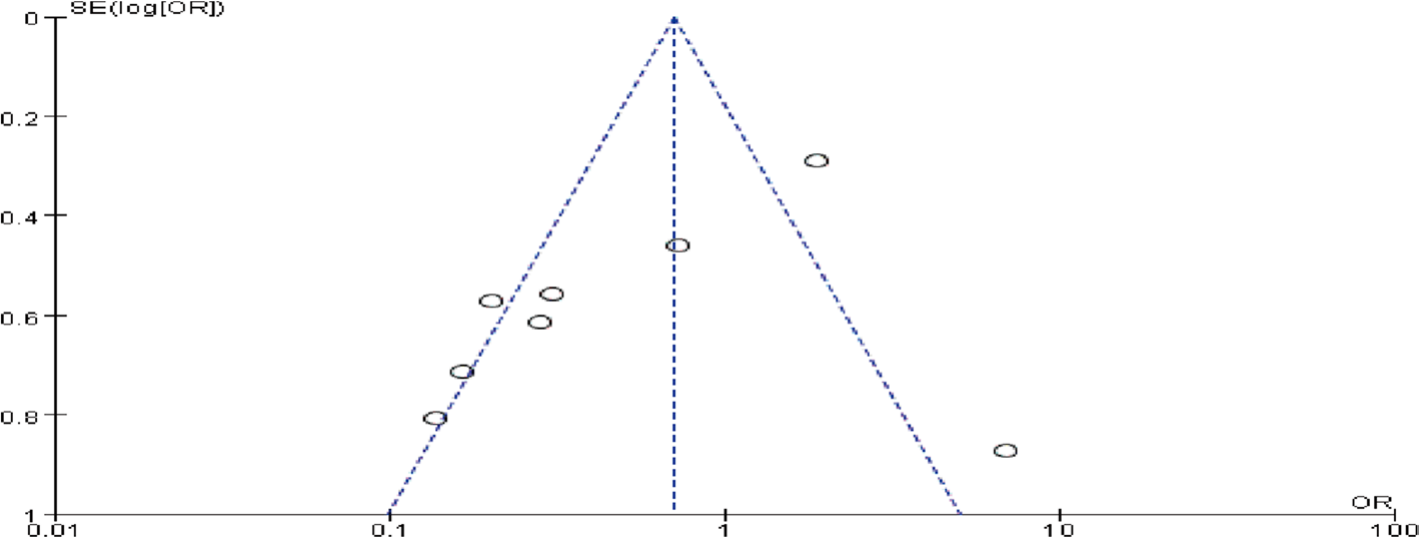
Funnel plot for ED/EA incidence from RevMan 5.4

**Fig. 5:**
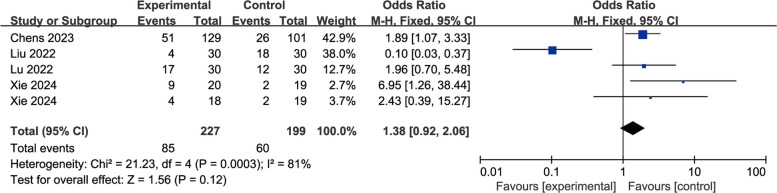
Subgroup analysis of high dose group VS control group on pediatric ED/EA (Fixed effects) from RevMan 5.4

**Fig. 6:**
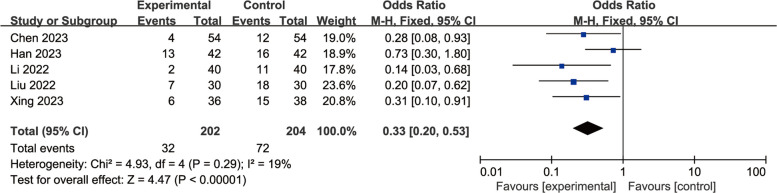
Subgroup analysis of low dose group VS control group on pediatric ED/EA (Fixed effects) from RevMan 5

**Fig. 7:**
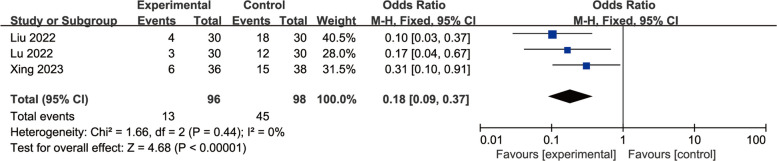
Esketamine VS control group on pediatric ED/EA of intranasal administration (Fixed effects) from RevMan 5.4

**Fig. 8:**
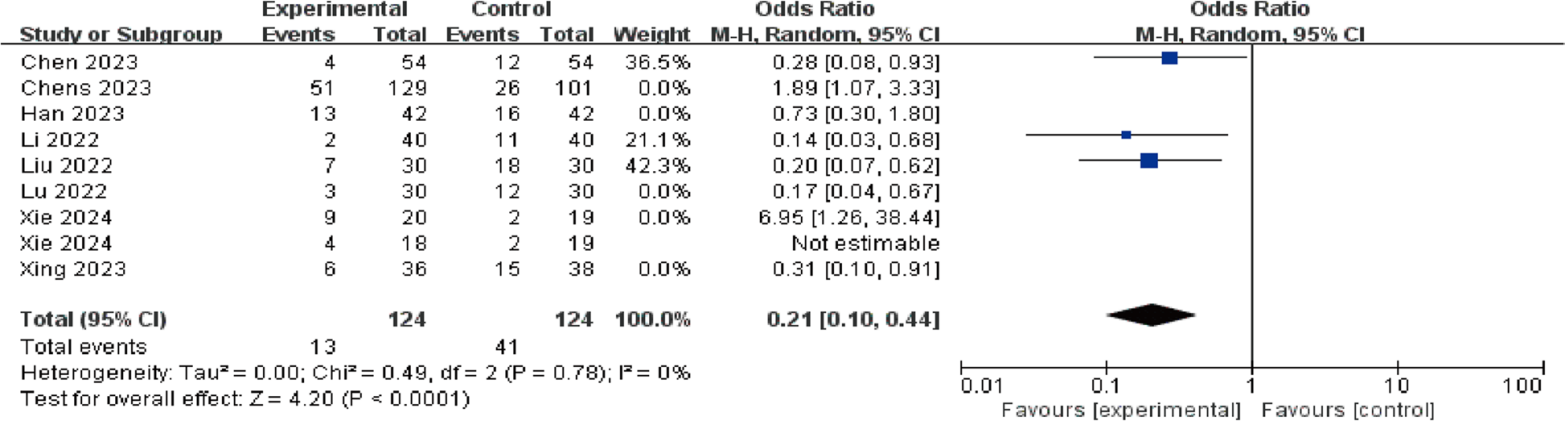
Subgroup analysis of esketamine VS placebo-controlled on pediatric ED/EA (Fixed effects) from RevMan 5.4

A total of eight studies were included to evaluate the effects of perioperative esketamine administration on postoperative ED/EA, as illustrated in Fig. 3. The overall findings indicated that the esketamine group exhibited a significant difference in the incidence of ED/EA among pediatric patients post-surgery compared to the control group [OR = 0.70, 95% CI: (0.51, 0.97), I^2^ = 80%, *P* for effect = 0.03]. Meanwhile, funnel plots and Begg's test (*P* = 0.17 > 0.05) were conducted for the primary outcome (ED/EA incidence), suggesting that the publication bias of this study was low and the results were relatively reliable (Fig. 4). High heterogeneity (I^2^ = 80%) likely reflects variability in dose, route, and study design. We conducted a subgroup analysis based on dosage, categorizing doses above 0.5 mg/kg as high and those below as low; results from the high-dose group did not reach statistical significance (OR = 1.38, 95% CI: (0.92, 2.06), I^2^ = 81%, *P* for effect = 0.12) (Fig. 5). Conversely, findings from the low-dose group suggested a significant difference [OR = 0.33, 95% CI: (0.20, 0.53), I^2^ = 19%, *P* for effect < 0.00001] (Fig. 6). Additionally, we performed a subgroup analysis based on different routes of administration; intranasal delivery demonstrated substantial significance [OR = 0.18, 95% CI: (0.09, 0.37), I^2^ = 0%, *P* for effect < 0 0.00001] (Fig. 7), whereas intravenous administration showed no statistically significant difference [OR = 0 0.91, 95%CI: (061, 1 0.36),I^2^ = 81%,P for effect = 0 0.06]. We also considered a subgroup analysis limited to a placebo-controlled study as shown in Fig. 8, where Esketamine can significantly reduce the incidence of ED/EA.

#### Esketamine for pain scores

**Fig. 9:**
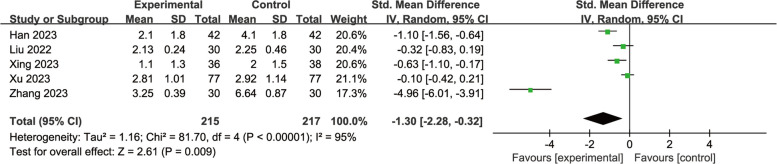
Comparison of pain scores between esketamine and control groups (Fixed effects) from RevMan 5.4

**Fig. 10:**
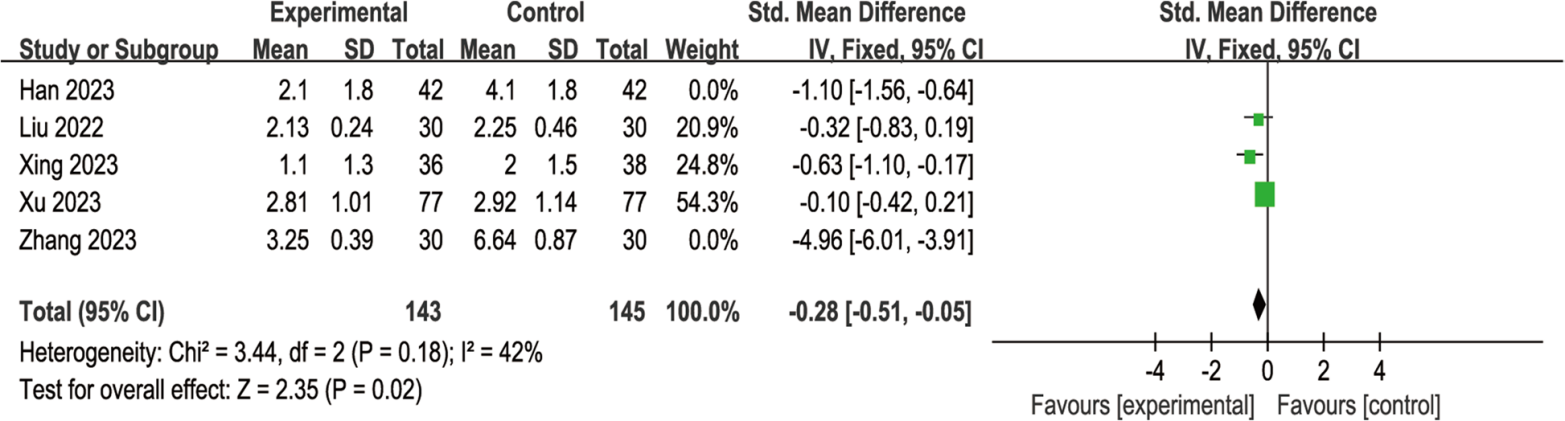
Pain scores between esketamine and control groups after excluding the identified outlier studies (Fixed effects) from RevMan 5.4

Among the eleven trials, only five original data were consistent with the log-normally distribution [10, 17, 19, 21, 22]. Our meta-analysis result showed significant differences, but high heterogeneity, as shown in Fig. 9 [SMD = −1.30, 95% CI: (−2.28, −0.32), I^2^ = 95%, P for effect = 0.009].When we removed the highly heterogeneous studies, the results are shown in Fig. 10, with I^2^ dropping from 95 to 42%.

#### Esketamine for PONV

**Fig. 11:**
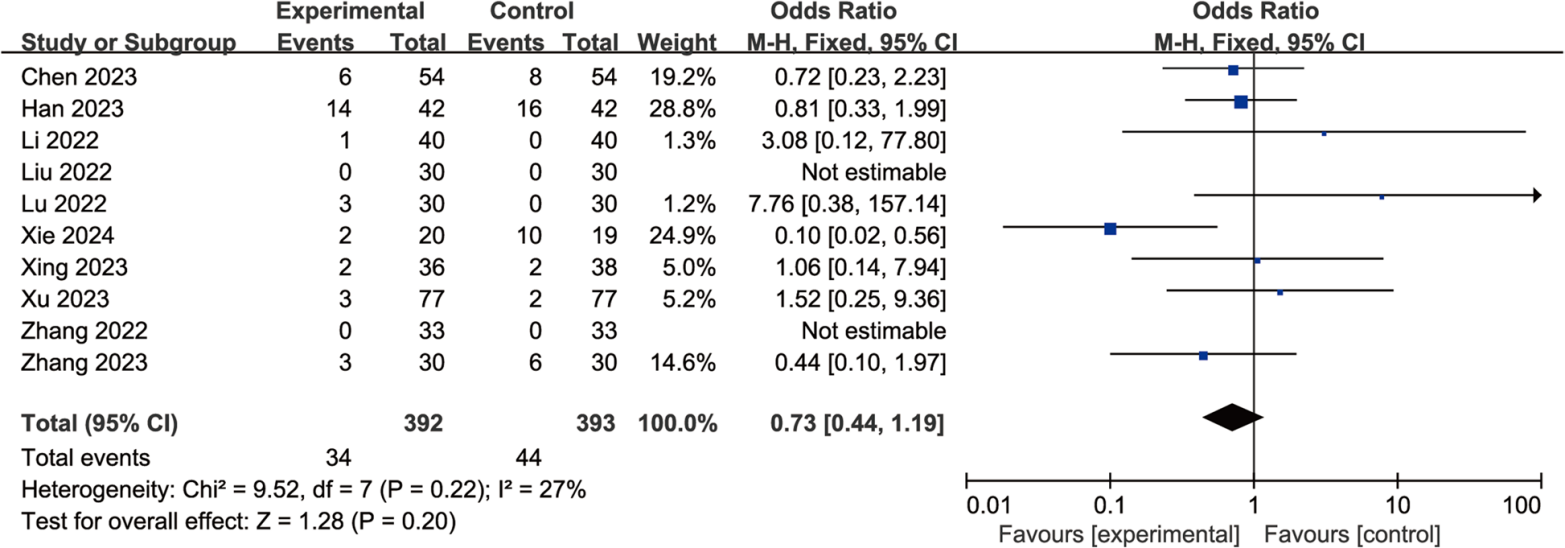
Comparison of PONV between esketamine and control groups (Fixed effects) from RevMan 5.4

We also conducted a meta-analysis on the effect of esketamine on postoperative PONV. There was only one report without postoperative PONV-related data [14], and ten trials were finally included for meta-analysis. Results As shown in Fig. 11, esketamine showed no significant difference in postoperative PONV compared with the control group [OR = 0.73, 95%CI: (0.44, 1.19), I^2^ = 27%, *P* for effect = 0.20].

### Sensitivity analysis

The sources of heterogeneity are typically assessed in relation to factors such as publication year, study methodology, geographical location, authorship, type of surgery, dosage and administration of esketamine, among others. However, we contend that the heterogeneity observed in this meta-analysis is predominantly influenced by the dosage and usage of esketamine. Consequently, Stata software was employed for meta-regression analysis. Notably, only the dosage factor yielded a *P* value below 0.05 (Supplementary Table 1). We also conducted a sensitivity analysis by sequentially excluding studies from our review. In the context of ED or EA affected by esketamine compared to the control group, studies [14, 20] emerged as significant contributors to heterogeneity; I2 decreased from 80 to 13% following their removal. For the subgroup examining esketamine's impact on postoperative pain scores relative to controls, studies [17, 23] were identified as primary sources of heterogeneity; I2 diminished from 95 to 42% after their exclusion. In high-dose esketamine subgroup analyses within intravenous administration contexts, I2 reduced from 81 to 0% upon removing study [10] and declined from 81 to 47% with the exclusion of study [14].

## Discussion

This meta-analysis incorporated totally 10 randomized controlled trials (RCTs) and one cohort study, aiming to evaluate the effects of esketamine on ED/EA, PONV, and pain scores in pediatric patients relative to the control group. The findings indicated that esketamine could effectively decrease the incidence of ED/EA in pediatric patients even though significant heterogeneity was observed. Subgroup analysis revealed that low-dose esketamine demonstrated more consistent results with lower heterogeneity. In addition, postoperative pain scores were shown to be lower in this population; however, the results exhibited considerable heterogeneity. Notably, esketamine did not increase the incidence of PONV when compared with the control group.

Currently, the underlying mechanisms of ED/EA in pediatric patients remain unknown. However, factors including inhalation of anesthetic agents (e.g., sevoflurane and isoflurane), age of the child, type of surgical procedure, pain levels, preoperative anxiety, and physiological conditions are all implicated [26–28, 29]. Notably, a recent meta-analysis has indicated that sevoflurane is associated with a higher risk of postoperative ED/EA compared to isoflurane [30]. Both pharmacological and non-pharmacological methods can be used for preventing the onset of ED/EA, with pharmacological options including esketamine, benzodiazepines, opioids, propofol, and dexmedetomidine among others [11, 12, 17, 31–33].

Meta-analysis has demonstrated that ketamine can decrease the incidence of ED/EA in children [34]. Therefore, does esketamine, as its enantiomer, also mitigate the occurrence of ED/EA in this population? Our findings indicate that this is indeed the case. However, similar to ketamine, there exists a significant degree of heterogeneity. The difference in control group drugs is also a source of high heterogeneity. When we only selected placebos as controls for inclusion in the meta-study, it was found that Esketamine could significantly reduce the incidence of ED/EA.Further subgroup analysis revealed that low-dose esketamine demonstrated more consistent results with lower heterogeneity in inhibiting ED/EA, consistent with a recent meta-analysis on pediatric gastroenteroscopy. This meta-analysis indicates that a single dose of ≤ 0.3 mg/kg can mitigate the side effects associated with gastroenteroscopy in children [35]. The Dixon and Massey up-and-down method was employed to determine the ED_50_ dose of esketamine for pediatric gastroscopy, which was shown to be 0.143 mg/kg [36]. Additionally, it was found that 0.3 mg/kg esketamine effectively lowered the PAED score without increasing the incidence of PONV in children [25]. Although doses exceeding 0.5 mg/kg are recommended for sedation/analgesia in children outside the operating room owing to their potential to decrease hypotension incidence, they may also cause increased side effects including dizziness [37]. In a recent pediatric dental procedure, it was determined that the ED_95_ dose of esketamine nasal spray reached 1.99 mg/kg. Despite this dosage significantly surpasses recommendations, several factors may explain this: firstly, administration via nasal spray; secondly, substantial stimulation and high pain intensity during dental procedures; and thirdly, relative ease of drug administration [38].

Esketamine can be administered through various different routes, including intravenous, nasal, and intramuscular injection. Transnasal administration of dexmedetomidine has been shown to reduce ED/EA in pediatric patients [39]. Similarly, our subgroup analysis corroborated that transnasal esketamine administration effectively diminished ED/EA in children. Perioperative anxiety is an independent risk factor for ED/EA post-surgery; thus, mitigating anxiety prior to surgery is vital for reducing these occurrences [40]. Nasal administration stands out as the most convenient and rapid method of delivery. Notably, a study performed by Liu et al. indicated that a dosage of 1 mg/kg via nasal spray was more effective in decreasing the incidence of ED/EA when compared with 0.5 mg/kg [10]. However, the combination of intranasal esketamine at 0.5 mg/kg with dexmedetomidine at 1 µg/kg resulted in a greater reduction in ED/EA than administering intranasal esketamine alone at 1 mg/kg [18]. As a result, optimal dosing varies across different routes of administration. Although high heterogeneity and lack of statistical significance were observed within the intravenous group during subgroup analysis, removing certain study [14] reversed these results primarily owing to this high heterogeneity.

In a meta-analysis examining tonsillectomy in pediatric patients, it was observed that ketamine significantly reduced the incidence of PONV when compared with the control group [41]; nevertheless, esketamine did not yield similar results when juxtaposed with the control group in our meta-analysis. Subsequent investigations revealed that PONV rates were lower in the ketamine cohort relative to those receiving opioids [42], whereas this trend was reversed in comparison with against dexmedetomidine [43]. Findings from an adult study devoid of opioid use indicated a significant decrease in PONV occurrences without opioid administration [24, 44, 45]. Consequently, there exists a substantial positive correlation between the utilization of opioid drugs and the incidence as well as severity of PONV.

Recent meta-analyses have substantiated that esketamine is both safe and effective, enhancing sedation and analgesia in pediatric patients outside the operating room [37]. Our own meta-analysis yielded similar findings. In a multicenter randomized controlled trial involving pediatric endoscopy, the esketamine group showed a significantly higher success rate for initial endoscopic placement relative to the nalbuphine group [13]; moreover, this conclusion was also corroborated in children with Autism Spectrum Disorder (ASD) [46]. Although our results reveal considerable heterogeneity, they remain consistent with prior studies.

In the current meta-analysis, esketamine exhibited significant heterogeneity in ED/EA (I^2^ = 80%) and pain scores (I^2^ = 95%) among children compared with the control group. Evidently, subgroup analysis serves as a vital method for addressing high heterogeneity [47]. It is proposed that potential sources of this high heterogeneity may include the age range of participants, variations in study methodologies, routes and dosages of administration, as well as inherent biases within the studies. Moreover, a meta-regression approach was used to identify these sources of heterogeneity; if the *P*-value from the meta-regression was < 0.05, subsequent subgroup analyses could be conducted for that factor [47, 48]. Our findings indicated that only dosage emerged with a *P*-value < 0.05 during meta-regression analysis; thus, we proceeded with a subgroup analysis based on dosage revealing that lower doses effectively lowered ED/EA occurrences in children. This has been consistently validated in clinical practice. In pediatric anesthesia, multiple combined agents are routinely administered to achieve optimal anesthetic conditions, while simultaneously minimizing adverse effects associated with the use of single-agent regimens. Despite obtaining a *P*-value greater than 0.05 for usage factors in our meta-regression, a separate meta-analysis focusing on different usages was still performed; clearly, I^2^ decreased to 47% within the intravenous administration group after excluding Study 12 from consideration. Furthermore, a case-by-case removal strategy was carried out to mitigate high heterogeneity [49]; following the exclusion of Studies [14, 20] from the ED/EA group and Studies [17, 22] from the pain scores group respectively, with I^2^values falling below 50%, The high heterogeneity is caused by the differences in surgical types, administration routes, and pain scales.

It is essential to elucidate the advantagesand limitations of our meta-analysis. We contend that the advantages are presented as follows: First, this represents the inaugural analysis of esketamine's effects on ED/EA in pediatric patients; Second, we have thoroughly examined the application of esketamine in children concerning its usage and dosage, which can thus provide a reference for future applications. Third, all results exhibiting high heterogeneity have been meticulously explored and interpreted, with an investigation into their sources. However, this meta-analysis is not without limitations: First, all studies from China, heterogeneity, limited sample size, differing definitions of ED/EA across studies, possible publication bias although Begg's test was non-significant. These meta-level findings may be particularly relevant to children of Asian descent; Second, the included studies lack comprehensiveness as they do not encompass unpublished research or studies lacking raw data.

## Conclusion

In conclusion, compared to the control group, esketamine significantly reduces the incidence of ED/EA in pediatric patients, with a notably higher evidence quality observed at lower doses. Furthermore, esketamine effectively alleviates postoperative pain scores in children without increasing the incidence of PONV.

## Supplementary Information

Supplementary Material 1.

Supplementary Material 2.

Supplementary Material 3.

## Data Availability

No datasets were generated or analysed during the current study.
