# Supplementary material for: The effect of esketamine on emergence delirium or agitation in children after anesthesia-a systematic review and meta-analysis
Source: BMC Anesthesiol. 2026 Mar 16;26:256. doi: 10.1186/s12871-026-03748-5 (PMC13104409; doi:10.1186/s12871-026-03748-5)
Supplement: Supplementary file 1 — Supplementary Material 1. [file 12871_2026_3748_MOESM1_ESM.docx]

**Author(s):** Yang Wei

**Question:** Esketamine compared to control group for ED/EA incidence

**Setting:** Pediatric anesthesia

**Bibliography:**

| **Certainty assessment** | | | | | | | **№ of patients** | | **Effect** | | **Certainty** | **Importance** |
| --- | --- | --- | --- | --- | --- | --- | --- | --- | --- | --- | --- | --- |
| **№ of studies** | **Study design** | **Risk of bias** | **Inconsistency** | **Indirectness** | **Imprecision** | **Other considerations** | **esketamine** | **control group** | **Relative (95% CI)** | **Absolute (95% CI)** |  |  |
| **esketamine for postoperative nausea and vomiting** | | | | | | | | | | | | |
| 10 | randomised trials | not serious | not serious | not serious | not serious |  | 34/392 (8.7%) | 44/393 (11.2%) | **OR 0.73** (0.44 to 1.19) | **28 fewer per 1,000** (from 59 fewer to 18 more) | - | CRITICAL |
| **esketamine for ED or EA** | | | | | | | | | | | | |
| 8 | randomised trials | not serious | serious^a^ | not serious | not serious |  | 95/381 (24.9%) | 112/354 (31.6%) | **OR 0.70** (0.51 to 0.97) | **72 fewer per 1,000** (from 125 fewer to 7 fewer) | -^a^ | CRITICAL |
| **esketamine for pain scores** | | | | | | | | | | | | |
| 3 | randomised trials | not serious | not serious | serious^b^ | not serious |  | 143 | 145 | - | SMD **0.28 lower** (0.51 lower to 0.05 lower) | -^b^ | CRITICAL |

**CI:** confidence interval; **OR:** odds ratio; **SMD:** standardised mean difference

#### Explanations

a. The drug dosage, type of surgery, placebo, etc. are inconsistent

b. The sample size is small
