# Supplementary material for: The effect of esketamine on emergence delirium or agitation in children after anesthesia-a systematic review and meta-analysis
Source: BMC Anesthesiol. 2026 Mar 16;26:256. doi: 10.1186/s12871-026-03748-5 (PMC13104409; doi:10.1186/s12871-026-03748-5)
Supplement: Supplementary file 3 — Supplementary Material 3. [file 12871_2026_3748_MOESM3_ESM.doc]

Meta-regression (esketamine versus placebo): estimate of between-study variance % residual variation due to heterogeneity.

|  | Coef. | Std. Err. | t | P>|t| | [95% Conf. Interval] | |
| --- | --- | --- | --- | --- | --- | --- |
| Usage of esketamine | .6628475 | .5833942 | 1.14 | 0.307 | -.836815 | 2.16251 |
| Dosage of esketamine | -1.286897 | .3285626 | -3.92 | 0.006 | -2.063824 | -.5099702 |
| Publication of year | .2805861 | .7296856 | 0.38 | 0.716 | -1.59513 | 2.156303 |
